# Evaluation of cardiac indices using M-mode echocardiography after administration of metoclopramide and ondansetron in donkeys (*Equus asinus*): an experimental study

**DOI:** 10.3389/fvets.2023.1189710

**Published:** 2023-08-23

**Authors:** Mohamed Marzok, Mahmoud Kandeel, Khaled Alkhodair, Sherief Abdel-Raheem, Hisham Ismail, Alshimaa Farag, Hossam Ibrahim, Maged El-Ashkar, Saad Shousha, Sabry El-Khodery

**Affiliations:** ^1^Department of Clinical Sciences, College of Veterinary Medicine, King Faisal University, Al-Ahsa, Saudi Arabia; ^2^Faculty of Veterinary Medicine, Department of Surgery, Kafrelsheikh University, Kafrelsheikh, Egypt; ^3^Department of Biomedical Sciences, College of Veterinary Medicine, King Faisal University, Al-Ahsa, Saudi Arabia; ^4^Faculty of Veterinary Medicine, Department of Pharmacology, Kafrelsheikh University, Kafrelsheikh, Egypt; ^5^Department of Anatomy, College of Veterinary Medicine, King Faisal University, Al-Ahsa, Saudi Arabia; ^6^Department of Public Health, College of Veterinary Medicine, King Faisal University, Al-Ahsa, Saudi Arabia; ^7^Faculty of Veterinary Medicine, Department of Animal Nutrition and Clinical Nutrition, Assiut University, Assiut, Egypt; ^8^Faculty of Veterinary Medicine, Department of Food Hygiene "Meat Hygiene", Assiut University, Assiut, Egypt; ^9^Faculty of Veterinary Medicine, Department of Internal Medicine, Infectious Diseases and Fish Diseases, Mansoura University, Manosura, Egypt; ^10^Faculty of Veterinary Medicine, Department of Physiology, Benha University, Benha, Egypt

**Keywords:** prokinetics, echocardiography, arrythmia, heart abbreviations, donkeys

## Abstract

The aim of the present study was to evaluate cardiac indices using M-mode echocardiography after the administration of metoclopramide and ondansetron in donkeys. For this purpose, 10 apparently healthy Egyptian Baladi donkeys (*Equus asinus*) were used in a crossover prospective study. Two trials were conducted with the administration of metoclopramide hydrochloride anhydrous at a dose of 0.25 mg Kg^−1^ and ondansetron hydrochloride sodium at a dose of 0.15 mg Kg^−1^. The control group (placebo) received a total volume of 50 mL of isotonic saline at 0.9%. An echocardiographic examination was performed using a Digital Color Doppler Ultrasound System equipped with a 2–3.9 MHz phased array sector scanner transducer. In general, the fractional shortening (FS%) was significantly affected by the time for metoclopramide (*p* = 0.031) and ondansetron (*p* = 0.047) compared with those of placebo, with treatment with metoclopramide provoking significantly higher percentages of FS% at T60 (*p* = 0.009) and T90 (*p* = 0.028) compared with those for ondansetron and placebo. The interaction of time x treatment also showed a statistically significant alteration of FS% (*p* < 0.05), while the values returned to the basal line at T240. Metoclopramide induced a significant decrease in E-point to septal separation (EPSS) at T90 (*p* = 0.005), and T240 (*p* = 0.007) compared with ondansetron and placebo. The time x treatment interaction also showed a significant (*p* < 0.05) variation in EPSS, with values returning to the basal line at T300. Mitral valve opening velocity (DE SLP) values were significantly affected by time (*p* = 0.004) in the metoclopramide group compared with those of ondansetron and placebo. Administration of metoclopramide and ondansetron provoked significant alterations of DE SLP at T60 (*p* = 0.039), T120 (*p* = 0.036), and T300 (*p* = 0.005) compared with placebo. In conclusion, caution should be exercised when administering both treatments, especially to animals with suspected cardiac problems.

## 1. Introduction

Gastrointestinal propulsive motility in the horse depends on a complex interaction between neural, hormonal, vascular, and neuromuscular pathways (1). Abnormal gastrointestinal propulsive motility (ileus) leads to stasis of the aboral passage of ingesta and is considered a common cause of mortality and morbidity in equines (2). Anesthesia, activation of inhibitory spinal and sympathetic reflexes, humoral agents, systemic shock, electrolyte disturbances, persistent luminal distention, ischemia, peritonitis, endotoxemia, and other inflammatory conditions have been reported to cause postoperative ileus (POI) in horses (3). It has been postulated that POI is attributed to several pathways, including loss of dissociation between electrical and mechanical activity, intrinsic or extrinsic electrical activity, and incoordination of contractile activity from regional stimuli (4).

Since the cause of paralytic ileus is likely to be multifactorial, various therapeutic agents have been used to target specific causes with variable success (1). Prokinetics are agents that facilitate or enhance the net movement of feed along the length of the intestinal tract and do not simply produce an uncoordinated increase in the local contractile activity (4). These agents act by enhancing the release of an excitatory neurotransmitter, overriding the inhibitory effect of a biologically active substance, or stimulating the release of a gastrointestinal hormone (5). Metoclopramide, a 5-HT4 agonist and 5-HT3 receptor antagonist, in addition to a dopamine D_2_ receptor antagonist, can suppress the vagal ganglia, leading to increased acetylcholine release at the nerve endings and promoting gastrointestinal motility (6–8). Continuous infusion of metoclopramide in horses with POI has restored coordinated gastroduodenal activity and gastrointestinal ingesta transit (9, 10). However, dopamine receptors have not been documented in the equine gastrointestinal tract (11). The clinical use of metoclopramide has also been evaluated in horses in different situations, such as after small-intestinal resection and anastomosis (10), in horses receiving endotoxin (12), gastric emptying, jejunal and cecal motility (8), and duodenitis/proximal jejunitis (DPJ) (13).

Ondansetron is considered a selective 5-HT3 serotonin receptor antagonist that acts both centrally (chemoreceptor trigger zone) and peripherally (acting on the vagus nerve terminals of the GIT) to control and reduce the frequency of vomiting in dogs and cats with frequent or severe vomiting (14) and in pancreatitis in animals (15) and cats with hepatic lipidosis (16).

With metoclopramide, the cardiovascular adverse effects are rare, but some can be fatal (17), while ondansetron can cause arrhythmias with either tachycardia or bradycardia, electrocardiographic alterations such as second-degree heart block, QT/QTc interval prolongation, ST segment depression, palpitations, and syncope (18). Rarely and predominantly with intravenous ondansetron, transient ECG changes that included QT interval prolongation have also been reported in subjects with electrolyte abnormalities (such as hypokalemia or hypomagnesemia), congestive heart failure, and bradyarrhythmias (19). Echocardiography has been shown to be effective in assessing cardiac dimensions and function in healthy and diseased donkeys (20, 21). There is limited data on the use of metoclopramide and ondansetron in donkeys. In addition, both medications’ side effects on cardiac functions remain to be fully elucidated. Therefore, the objective of the present study was to investigate the adverse effects of metoclopramide and ondansetron on cardiac functions in donkeys using M-mode echocardiography. We hypothesized that both drugs may have a potential side effect on the cardiac functions of donkeys and that echocardiography may help detect such alterations.

## 2. Materials and methods

### 2.1. Animals

A total of 10 apparently healthy Egyptian Baladi donkeys (*Equus asinus*) were used in a crossover prospective study. The donkeys ranged in age from 5 to 9 years (mean ± standard deviation, 6.7 ± 1.4 years), and in body weight from 100 to 160 kg (mean ± standard deviation, 125.5 ± 19.6 kg). All donkeys were selected to be clinically healthy and free of any cardiovascular disease based on complete clinical and cardiac examinations, which included assessment of the heart rate, regularity of the heartbeats, strength of the heart sounds, abnormal heart sounds, and electrocardiography (22). Therefore, any systemic disease, tachycardia or bradycardia, cardiac arrhythmia, cardiac murmur, detectable cardiac defects, and those donkeys that were not compliant with handling were considered exclusion criteria. Two weeks before the commencement of the study, donkeys were stabled separately in straw-bedded stalls and fed 1 kg of hay /100 kg and 0.5 kg of concentrate twice a day with free access to water. This study was carried out at the Department of Internal Medicine and Infectious Diseases, Faculty of Veterinary Medicine, Mansoura University, Egypt. The study was approved by the Animal Welfare and Ethics Committee, Faculty of Veterinary Medicine, Mansoura University (Code: VET.03.12.19), Egypt.

### 2.2. Study design

A crossover prospective experimental study included two treatment trials and one control study with five donkeys each. The first two trials included the administration of metoclopramide hydrochloride anhydrous (Primperan 10 mg/2 mL solution, SANOFI, Egypt) at a dose of 0.25 mg/kg BW (11) and ondansetron hydrochloride sodium (Zofran 8 mg/4 mL solution, GlaxoSmithKline Pakistan Ltd.) at a dose of 0.15 mg/kg BW (23). Both medications were administered intravenously in an equal volume of 50 mL of sterile isotonic sodium chloride 0.9%. For the control group (placebo), a total volume of 50 mL of isotonic saline 0.9% (EL-Nasr pharmaceutical Chemicals Co, Egypt) was also administered via intravenous injection.

According to the pharmacokinetics of metoclopramide (24) and ondansetron (25) in horses, the interval between the two treatments was 1 week. Echocardiographic examination and cardiac indices were evaluated for each donkey before treatment (T0) and at T15, T30, T60, T90, T120, T180, T240, T300, and T360 minutes after treatment administration. Evidence of clinical side effects, such as muscle tremors, the inability to stand on legs, and shifting from one limb to another, were all observed, and the findings were documented simultaneously. In general, access to food and water was not allowed during the monitoring periods.

### 2.3. Echocardiographic examination

Transcutaneous echocardiographic examinations were performed according to the standard methods described by Youssef et al. (21). All echocardiographic procedures and precautions were followed according to the recommendations of the American Society of Echocardiography. Echocardiographic examinations were performed with a CHISON Digital Color Doppler Ultrasound System, iVis 60 EXPERT VET (CHISON Medical Imaging Co., Ltd., Wuxi, China), using a 2–3.9 MHz phased array transducer with a maximum depth of 24.1 cm. The M-mode echocardiographic evaluation of the heart was guided by the simultaneous display of real-time two-dimensional (2-D) echocardiographic images.

### 2.4. Echocardiographic measurements

Echocardiographic examinations were performed using the leading-edge method according to the recommendations of the American Society of Echocardiography from frozen images on the screen (26). Guided two-dimensional images were included, the right-parasternal long-axis 4-chamber view (4C) and the right-parasternal long-axis view of the left ventricular outflow tract (LVOT). M-mode images were obtained from the right-parasternal short-axis view of the left ventricle (LV) at the chordal level (27). M-mode variables measured at the chordal level included inter-ventricular septal thickness at end-systole (IVSs), interventricular septal thickness at end-diastole (IVSd), left ventricular internal diameter at end-systole (LVIDs), left ventricular internal diameter at end-diastole (LVIDd), left ventricular posterior wall thickness at end-systole (LVPWs), and left ventricular posterior wall thickness at end-diastole (LVPWd) were assessed using the Cube Method. Meanwhile, the Teicholz formula (28) was used to calculate the left ventricular volume at end-systole (LVESV) and end-diastole (LVEDV). Other parameters, namely fractional shortening (FS), ejection fraction (EF), and stroke volume (SV) were calculated using established formulas suggested by Kienle (29). Measurements in the 4C view included the left atrial diameter (LAD) at the mitral valve annulus and the maximum dimension parallel to the mitral valve annulus (27). With the cursor line positioned perpendicularly over the mitral valves (MVs), two leaflets were identified that produced an inverted M-shaped image on the M-mode. The following measurements were recorded: DE slope (mitral valve opening speed), EF slope (mitral valve closing velocity), E-point to septal separation (EPSS), and IRT (isovolumic relaxation time).

In the LVOT view, by slightly pointing the transducer more anteriorly and toward the leaflet, the aorta and left atrium were measured. Similarly, the aortic diameter (AOD) at the level of the sinotubular junction, the aortic valve diameter (AVD), the left ventricular ejection time (LVET), and the right ventricular outflow tract diameter (RVOTD) and be measure (27).

### 2.5. Statistical analysis

Data analysis was performed using a commercial statistical software program (SPSS for Windows, version 21; SPSS Inc., Chicago, IL, United States). The data related to explanatory variables (treatments) and response variables (cardiac indices) were identified and sorted. Data were tested for normality of distribution using the Shapiro–Wilk test. According to the results of the Shapiro–Wilk test, the data were not normally distributed, so the Kruskal-Wallis test was used to compare placebo, metoclopramide, and ondansetron. The median, minimum, and maximum values were calculated for each variable, respectively. Multiple comparisons between statistically different groups were performed using the Mann–Whitney test, which was used to assess within-group differences and evidence of a time x treatment interaction. The Friedman test was used to assess the effect of treatment at different time points. For all statistical analyses, differences at *p* < 0.05 were considered significant.

## 3. Results

**Figure 1:**
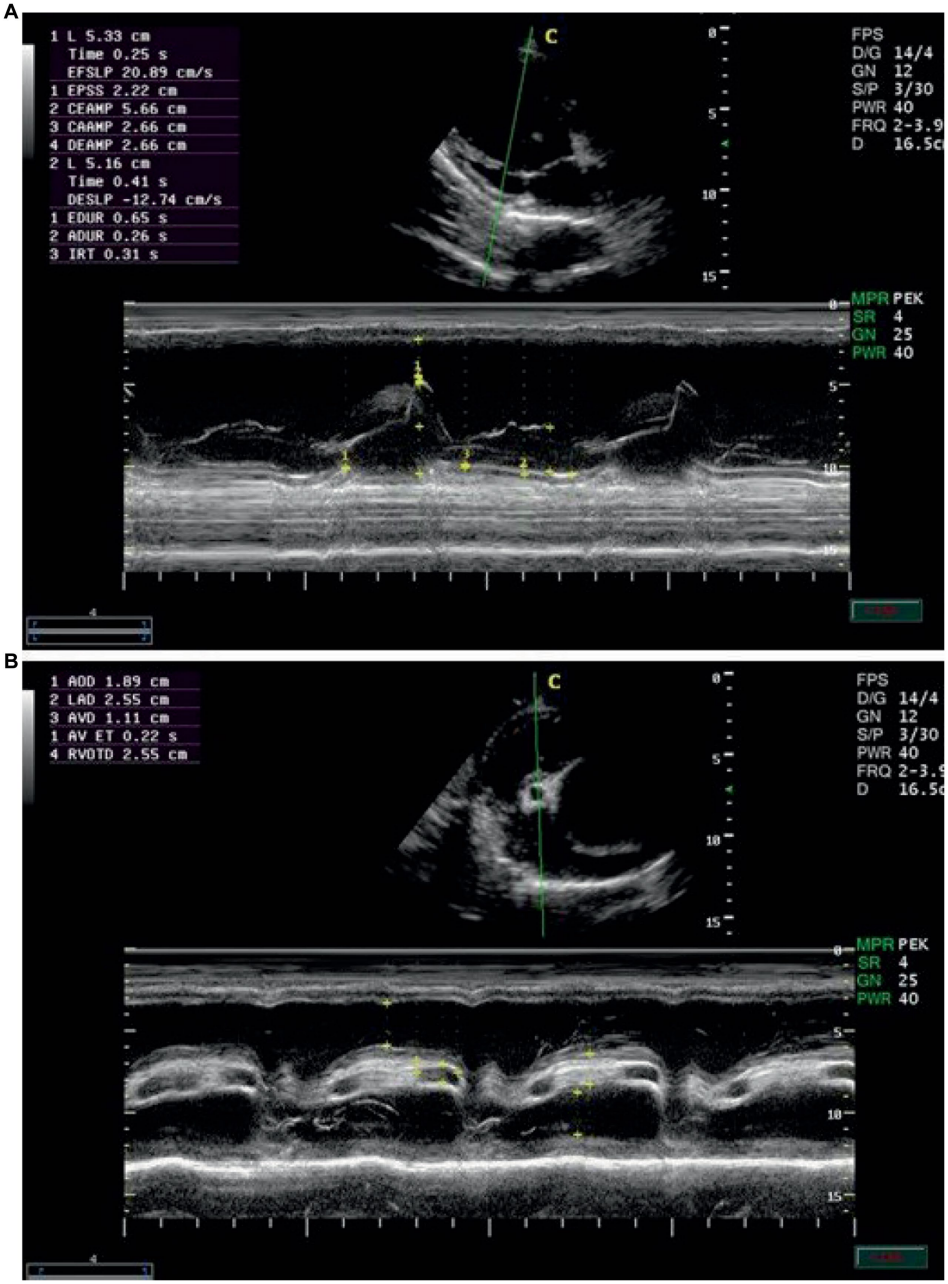
**(A)** The right parasternal long-axis view of the left ventricular outflow tract in examined donkeys using B-mode guidance for M-mode echocardiography. **(B)** The right parasternal view of the long-axis left ventricular outflow tract in examined donkeys using M-mode echocardiography at zero time. IVS = interventricular septum; LVID = left ventricular internal diameter; LVPW = left ventricular posterior wall.

**Figure 2:**
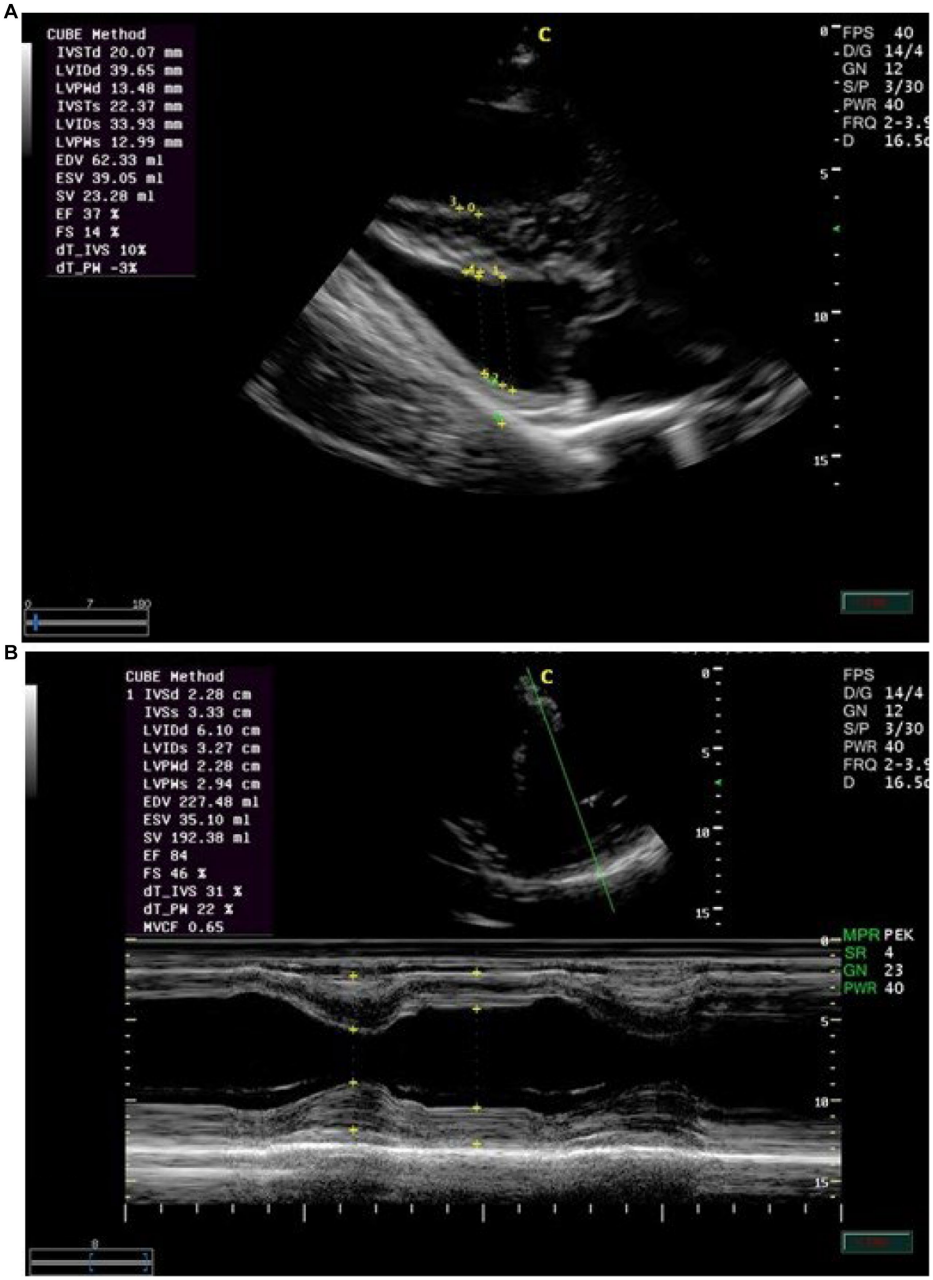
**(A)** The right parasternal long-axis view of the left ventricular outflow tract of the mitral valve in examined donkeys using M-mode echocardiography at zero time. IVS = interventricular septum; LV = left ventricle; LVW = left ventricular wall; MV = mitral valve. **(B)** The right parasternal long-axis view of the left ventricular outflow tract view of the aortic valve in examined donkeys using M-mode echocardiography at zero time. RA = right atrium; AO = aorta; AOV = aortic valve; LA = left atrium.

Echocardiographic findings were recorded to assess the left ventricular images (Figures 1A,B), mitral valve (Figure 2A), and aortic valve (Figure 2B) images in the examined donkeys. The effects of time, treatment, and time x treatment interaction after administration of metoclopramide and ondansetron in the investigated donkeys are presented in Tables 1–5. In general, FS% was significantly affected by the time for metoclopramide (*p* = 0.031) and ondansetron (*p* = 0.047) compared with placebo. However, treatment with metoclopramide provoked significantly higher percentages of FS at T60 (*p* = 0.009) and T90 (*p* = 0.028) compared with those of ondansetron and placebo. The time x treatment interaction also showed statistically significant alterations of FS% (*p* < 0.05), while values returned to the basal line at T240 (Table 1). Other cardiac dimensions, namely IVS, LVID, and LVPW at end-diastole and end-systole, EDV, ESV, and SV, and cardiac indices such as EF, showed no significant (*p* > 0.05) variation among the examined donkeys.

**Table 1:** Fractional shortening (%) [median (minimum-maximum)] after administration of metoclopramide and ondansetron in donkeys at different time points.

Treatment time after administration (min)										
	T0	T15	T30	T60	T90	T120	T180	T240	T300	T360
Placebo	35 (31–45)	36 (30–46)	37 (31–46)	36 (30–45)^a^	37 (31–45)^a^	37 (30–45)	36 (32–44)	36 (31–45)	35 (30–44)	36 (32–44)
Metoclopramide 0.25 mg Kg^−1^	36 (30–46)	38 (33–51)	42 (37–73)^*^	46 (40–66)^*b^	45 (37–56)^*b^	41 (34–49)^*^	45 (34–74)^*^	39 (29–46)	36 (32–61)	35 (25–53)
Ondansetron 0.15 mg Kg^-1 (*n* = 10)^	37 (31–46)	35 (27–45)	43 (33–56)^*^	43 (39–55)^*a^	40 (34–51)^*a^	43 (33–54)^*^	44 (22–55)^*^	38 (28–54)	36 (31–55)	35 (25–39)

^a,b^ Variables with different superscripts within the same time point significantly differ at *p* < 0.05.

Variables with (*) significantly differ at *p* < 0.05 over time.

**Table 2:** EPSS (distance between E-point of the mitral valve and IVS: interventricular septum) (cm) [median (minimum-maximum)] after administration of metoclopramide and ondansetron in donkeys at different time points.

Treatment	Time after administration (min)									
	T0	T15	T30	T60	T90	T120	T180	T240	T300	T360
Placebo	2.8 (1.9–4.8)	3 (2–4.8)	2.9 (1.9–4.7)	2.9 (1.9–4.8)	2.9 (1.8–4.7)^a^	3 (2–4.8)	2.9 (1.9–4.8)	2.9 (1.9–4.9)^a^	2.9 (2–4.8)	2.8 (1.9–4.9)
Metoclopramide 0.25 mg Kg^−1^ (*n* = 10)	2.9 (1.9–4.8)	2.8 (1.6–5.8)	2.7 (2.2–8.1)^*^	2.3 (0.9–8.5)^*^	2 (1.3–2.4)^b^	2.2 (1.1–3.1)	2.1 (1.4–3.9)	1.6 (1–2.8)^b^	2.7 (1.9–4.3)	2.8 (2.2–4.8)
Ondansetron 0.15 mg Kg^−1^ (*n* = 10)	2.9 (2–4.8)	3.2 (2.8–3.3)	3.3 (1.9–4.3)	3.4 (2.2–3.6)	2.5 (2.1–3.8)^a^	2.4 (2.2–3.8)	2.8 (2.1–3.4)	2.4 (2–3.2)^a^	3.1 (2–3.9)	3 (2.3–3.1)

^a,b^ Variables with different superscript letters within the same time point significantly differ at *p* < 0.05.

Variables with (*) significantly differ at *p* < 0.05 over time.

**Table 3:** EF SLP; Mitral valve closing speed (cm/s) [median (minimum-maximum)] after administration of metoclopramide and ondansetron in donkeys at different time points.

Treatment	Time after administration (min)									
	T0	T15	T30	T60	T90	T120	T180	T240	T300	T360
Placebo	6.9 (5.2–7.6)	7 (5.2–7.5)	6.9 (5.2–7.6)^a^	6.4 (5.2–7.7)^a^	6.8 (5.2–7.6)^a^	6.9 (5.2–7.5)^a^	6.9 (5.2–7.6)^a^	6.9 (5.2–7.6)^a^	6.9 (5.2–7.5)	7 (5.2–7.6)
Metoclopramide 0.25 mg Kg^−1^ (*n* = 10)	7 (5.2–7.5)	6 (5.3–7.8)	11.6 (7.3–15.8)^*b^	9.3 (6.9–9.8)^*b^	11.6 (7.6–12)^*b^	12.3 (10–15.2)^*b^	11.3 (7.5–15.3)^*b^	10.9 (8.6–17.3)^*b^	7.1 (7–7.3)	7.2 (7–7.5)
Ondansetron 0.15 mg Kg^−1^ (*n* = 10)	6.9 (5.2–7.2)	6.8 (5.8–7.2)	10.4 (8.6–11.9)^*b^	10.2 (8.9–10.6)^*ab^	11.5 (9.9–13.9)^*b^	11.6 (10–12.7)^*b^	11.6 (10–12.8)^*b^	11.2 (10–12.9)^*b^	7.1 (6–7.3)	7 (6.2–8)

^a,b^ Variables with different superscript letters within the same time point significantly differ at *p* < 0.05.

Variables with (*) significantly differ at *p* < 0.05 over time.

**Table 4:** DE SLP; Mitral valve opening speed (cm/s) [median (minimum-maximum)] after administration of metoclopramide and ondansetron in donkeys at different time points.

Treatment	Time after administration (min)									
	T0	T15	T30	T60	T90	T120	T180	T240	T300	T360
Placebo	9.2 (5.3–11.6)	9.1 (5.3–11.6)	9.5 (5.3–11.6)	9.4 (5.3–11.6)^a^	9.5 (5.4–1.6)	9.1 (5.5–11.6)^a^	9.3 (5.3–11.6)	9.2 (5.3–11.6)	9.1 (5.3–11.6)^a^	9.5 (5.3–11.9)
Metoclopramide 0.25 mg Kg^−1^ (*n* = 10)	9.1 (5.3–11.6)	10 (8.1–13.9)^*^	11.6 (6.7–13.4)^*^	10 (9.3–13)^*a^	10.6 (9–3.1)^*^	11.2 (10–4.9)^*b^	11.1 (9.6–2.7)^*^	11.4 (4.8–12.2)^*^	12 (9.2–16.5)^*b^	9.2 (6.7–12.6)^*^
Ondansetron 0.15 mg Kg^−1^ (*n* = 10)	9.5 (5.3–11.6)	10.2 (8.7–13.4)	10.4 (7.2–11.5)	11.6 (10–2.8)^ab^	10 (9.3–11.4)	10.2 (7.6–11.9)^a^	11.5 (10.2–12)	10.6 (8.4–12.6)	10.9 (10–12.2)^ab^	10.6 (7.2–12.8)

^a,b^ Variables with different superscript letters within the same time point significantly differ at *p* < 0.05.

Variables with (*) significantly differ at *p* < 0.05 over time.

**Table 5:** ET (Ejection time (seconds)) [median (minimum-maximum)] after administration of metoclopramide and ondansetron in donkeys at different time points.

Treatment	Time after administration (min)									
	T0	T15	T30	T60	T90	T120	T180	T240	T300	T360
Placebo	0.7 (0.2–0.7)	0.7 (0.2–0.7)	0.6 (0.2–0.7)^a^	0.6 (0.2–0.7)	0.7 (0.2–0.7)^a^	0.7 (0.2–0.7)^a^	0.6 (0.2–0.7)^a^	0.7 (0.2–0.7)	0.7 (0.1–0.7)	0.7 (0.2–0.7)
Metoclopramide 0.25 mg Kg^−1^ (*n* = 10)	0.7 (0.2–0.7)	0.5 (0.1–0.6)	0.4 (0.3–0.7)^*a^	0.3 (0.2–0.5)^*^	0.3 (0.1–0.6)^*b^	0.3 (0.2–0.4)^*b^	0.4 (0.3–0.7)^*a^	0.4 (0.2–0.4)^*^	0.5 (0.4–0.8)	0.7 (0.2–0.7)
Ondansetron 0.15 mg Kg^−1^ (*n* = 10)	0.6 (0.1–0.7)	0.4 (0.2–0.6)	0.3 (0.1–0.4)^*b^	0.3 (0.1–0.6)^*^	0.2 (0.1–0.3)^*b^	0.1 (0.1–0.8)^*b^	0.2 (0.1–0.5)^*b^	0.3 (0.2–0.7)^*^	0.4 (0.2–0.7)	0.7 (0.2–0.7)

^a, b^ Variables with different superscript letters within the same time point significantly differ at *p* < 0.05.

Variables with (*) significantly differ at *p* < 0.05 over time.

The values of EPSS were significantly affected by time (*p* = 0.003) and treatment with metoclopramide, being lower at T90 (*p* = 0.005) and T240 (*p* = 0.007) than those of ondansetron and placebo. In contrast, time had no significant effect after ondansetron administration (*p* = 0.209). Time x treatment also showed a significant (*p* < 0.05) variation in EPSS, with values returning to the basal line at T300 (Table 2). EF SLP values were significantly affected by time (*p* = 0.001), and treatment with metoclopramide and ondansetron at T30-T240 were higher than those of placebo. The time x treatment interaction showed a significant effect (*p* < 0.05), and the EF SLP values returned to basal values at T300 (Table 3).

DE SLP values were significantly affected by time (*p* = 0.004) in the metoclopramide group compared with those of ondansetron and placebo. Metoclopramide and ondansetron administration provoked significant alterations at T60 (*p* = 0.039), T120 (*p* = 0.036), and T300 (*p* = 0.005) compared with placebo. Metoclopramide produced higher values of DE SLP at T120 and T300 than did ondansetron, but the latter produced higher values of DE SLP at T60 and T180 compared with metoclopramide. Time x treatment showed a significant variation (*p* < 0.05) at T15 and continued until T360 (Table 4). Other mitral valve parameters, such as IRT, showed no significant (*p* > 0.05) variation between groups.

Ejection time was significantly influenced by the time after administration of metoclopramide (*p* = 0.029) and ondansetron (*p* = 0.010). ET values were significantly decreased at T30 (*p* = 0.020), T90 (*p* = 0.005), T120 (*p* = 0.046), and T180 (*p* = 0.010) after administration of metoclopramide and ondansetron compared with placebo. ET values were lower in the ondansetron group than in the metoclopramide group. Time x treatment interactions produced a significant variation (*p* < 0.05), but ET values returned to the basal line at T300 (Table 5). Other echocardiographic parameters from aortic valve images, namely LAD, AOD, AVD, and RVOTD, showed no significant (*p* > 0.05) alterations between groups.

## 4. Discussion

Disturbed intestinal motility patterns in equines are major clinical problems occurring due to several causes and can lead to intestinal diseases and clinical signs, which are to be encountered by the equine practitioner (2). Consequently, numerous investigations are aimed at the better development of therapeutic agents capable of modifying these motility patterns with minimal deleterious adverse effects. The present study compared the adverse effects of metoclopramide and ondansetron on cardiac indices in donkeys. To date, data regarding the use of ondansetron as a gastrointestinal promotility agent in equine patients is still scarce. The potential adverse cardiac effects are yet to be elucidated. Our findings demonstrated that the administration of metoclopramide and ondansetron provoked a significant elevation of FS% compared with those of placebo, with significantly higher values recorded with the administration of metoclopramide than with ondansetron, suggesting a state of altered left ventricular function that could be attributed to an impairment of the contractile and pumping functions of the heart. In addition, the increase in left ventricular diastolic size could stretch the myofibers and consequently increase fractional shortening (30). Our findings were consistent with previous studies (17). The authors found that metoclopramide can induce dose-dependent adverse cardiac effects, such as lengthening the electrocardiographic QT interval, syncopal episodes, and ventricular dysrhythmias. It has also been reported that metoclopramide induces torsade de pointes (“twisting of the peaks”), a specific type of abnormal cardiac arrhythmia, and sudden death (31). On the other hand, the use of ondansetron has caused prolongation of the QT interval on the ECG and induced arrhythmias in human patients (32). A similarly high prevalence of QT prolongation has been associated with the severity of left ventricular systolic dysfunction in humans with heart failure and ventricular arrhythmias (33).

Administration of metoclopramide produced a significantly lower value of EPSS at T90 and T240 compared with ondansetron. Such a decrease in EPSS values could be attributed to left ventricular filling and function. It has been noted that EPSS is a variable that may be useful in determining the presence or absence of severely depressed left ventricular systolic function (34). Thus, EPSS and FS% measurements had a moderately negative correlation.

The administration of metoclopramide and ondansetron significantly increased EF SLP and DE SLP in addition to ET at different time points. In general, alterations in EF SLP, DE, and SLP indicate left ventricular emptying and/or filling, while the decrease in ET could suggest left ventricular systolic dysfunction. In previous studies, it has been suggested that the decrease in ET may be associated with various diseases, such as valvular disease, coronary artery disease, arterial hypertension, or atrial fibrillation (35). It has also been shown that left ventricular ET depends on intrinsic contractility and is influenced by positive and negative inotropes, left ventricular failure, reduced afterload, and increased preload (35). The shortening of ET in left ventricular dysfunction is more complex. In the later study, the authors found a delay in the onset of ejection time in cases of left ventricular failure, while the velocity of myocardial fiber shortening is often reduced.

## 5. Conclusion

The results of the present study indicate that both metoclopramide and ondansetron adversely affect echocardiographic parameters in donkeys. Caution should be exercised when administering either treatment, especially to animals with suspected cardiac problems. However, the findings of this study in healthy donkeys may not be extrapolated to equines with cardiac diseases. Consequently, further studies are needed to determine whether the adverse effects of these treatments are related to clinical practice.

## Data availability statement

The raw data supporting the conclusions of this article will be made available by the authors, without undue reservation.

## Ethics statement

The animal studies were approved by the Animal Welfare and Ethics Committee, Faculty of Veterinary Medicine, Mansoura University (Code: VET.03.12.19). The studies were conducted in accordance with the local legislation and institutional requirements. Written informed consent was obtained from the owners for the participation of their animals in this study.

## Author contributions

MM, SS, HeI, KA, and MK: conceptualization. ME-A, SE-K, and SA-R: methodology. HuI and ME-A: software. MM, SE-K, and KA: validation. SE-K and MM: formal analysis. MM and MK: investigation and visualization. HeI, KA, and HuI: resources. HuI, SA-R, and SS: data curation. MM: writing—original draft preparation, project administration, and funding acquisition. SE-K, ME-A, and KA: writing—review and editing. MM and KA: supervision. All authors have read and agreed to the published version of the manuscript.

## Funding

This work was supported by the Annual Funding track of the Deanship of Scientific Research, Vice Presidency for Graduate Studies and Scientific Research, King Faisal University, Saudi Arabia (Grant Number No. 3110).

## Conflict of interest

The authors declare that the research was conducted in the absence of any commercial or financial relationship that could be construed as a potential conflict of interest.

## Publisher’s note

All claims expressed in this article are solely those of the authors and do not necessarily represent those of their affiliated organizations, or those of the publisher, the editors and the reviewers. Any product that may be evaluated in this article, or claim that may be made by its manufacturer, is not guaranteed or endorsed by the publisher.

## Figures and Tables

**Table:** 

IVS	Interventricular septum
LVPW	Left ventricular posterior wall
LVID	Left ventricular internal diameter
EDV	End diastolic volume
ESV	End systolic volume
FS	Fractional shortening
EF	Ejection fraction
SV	Stroke volume
DE slope of MVs	Mitral valve opening velocity
EF slope of MVs	Mitral valve closing velocity
EPSS	E-point to septal separation
IRT	Isovolumic relaxation time
LAD	Left atrial diameter
AOD	Aortic diameter
AVD	Aortic valve diameter
LVET	Left ventricular ejection time
RVOTD	Right ventricular outflow tract diameter
